# Fast sparse fractal image compression

**DOI:** 10.1371/journal.pone.0184408

**Published:** 2017-09-08

**Authors:** Jianji Wang, Pei Chen, Bao Xi, Jianyi Liu, Yi Zhang, Shujian Yu

**Affiliations:** 1 Institute of Artificial Intelligence and Robotics, Xi’an Jiaotong University, Xi’an, Shaanxi, China; 2 National Engineering Laboratory for Visual Information Processing and Applications, Xi’an Jiaotong University, Xi’an, Shaanxi, China; 3 Institute of Automation, Chinese Academy of Science, Beijing, China; 4 College of Mechanical and Electronic Engineering, Shandong University of Science and Technology, Qingdao, Shandong, China; 5 Computational NeuroEngineering Laboratory, University of Florida, Gainesville, Florida, United States of America; Soochow University, CHINA

## Abstract

As a structure-based image compression technology, fractal image compression (FIC) has been applied not only in image coding but also in many important image processing algorithms. However, two main bottlenecks restrained the develop and application of FIC for a long time. First, the encoding phase of FIC is time-consuming. Second, the quality of the reconstructed images for some images which have low structure-similarity is usually unacceptable. Based on the absolute value of Pearson’s correlation coefficient (APCC), we had proposed an accelerating method to significantly speed up the encoding of FIC. In this paper, we make use of the sparse searching strategy to greatly improve the quality of the reconstructed images in FIC. We call it the sparse fractal image compression (SFIC). Furthermore, we combine both the APCC-based accelerating method and the sparse searching strategy to propose the fast sparse fractal image compression (FSFIC), which can effectively improve the two main bottlenecks of FIC. The experimental results show that the proposed algorithm greatly improves both the efficiency and effectiveness of FIC.

## Introduction

Fractal image compression (FIC) is an image coding technology based on the local affine similarity of image structure. Since the original fractal image compression scheme was proposed by Barnsley and Jacquin [1, 2], FIC technology had demonstrated rapid development. Aside from the application as an image compression technology, FIC has been widely used in some other fields, such as image denoising [3, 4], image encryption [5, 6], image sharpening [7], and facial image recognition [8]. To date, FIC is still one of the research hotspots in image processing.

Although great progress had been made, FIC suffered from two important bottlenecks in the past two decades. One bottleneck is the overlong encoding time, and the other is that the image quality of the reconstructed images for some images with low structural similarity is usually unacceptable.

The overlong encoding time is an important problem of FIC, and most of the publications about FIC are focused on this topic [9–13]. Based on the fact that the affine similarity in FIC is equivalent to Pearson’s absolute correlation [14, 15], we proposed the absolute value of Pearson’s correlation coefficient (APCC) -based accelerating algorithms [16], which significantly speed up the encoding phase of FIC while preserving the reconstructed image quality well. For example, the APCC-based method only takes 0.0766 seconds to encode a 512 × 512 image on a home computer with CPU I3–2100 and windows XP OS, and the peak signal to noise ratio (PSNR) of the reconstructed image only degrades about 0.3 to 0.4 dB than that of the baseline fractal image compression (BFIC).

The other main bottleneck FIC technology facing is the image quality problem. For some images which have less structure-similarity, the reconstructed image quality of FIC is usually unacceptable. Many researchers also made their efforts to improve the reconstructed image quality of FIC [17–20]. One natural method to improve image quality is to reduce the size of range blocks [17]. For example, when the size of the range blocks is partitioned as 4 × 4, the reconstructed image quality is better than that of the range size 8 × 8. Decreasing the step-length between two adjoining domain blocks is another method [2], which improves the reconstructed image quality by enlarging the domain pool. Besides, Tong and Pi proposed a hybrid fractal image compression scheme with linear predictive coding in 2003 [18], which yielded better compression quality at the same compression ratio. By integrating partitioned iterated function system and linear transforms, Nappi *et al* enhanced both subjective and objective image quality in 2007 [19]. Wang *et al* proposed a SSIM-based scheme in 2011 [20], in which the reconstructed image quality is more appropriate for human visual system (HVS).

Though many methods were proposed to improve the reconstructed image quality of FIC, there were still a lot of works left. For example, when the range size is getting smaller, the convergence of decoding becomes weak. Moreover, most of the schemes improve the image quality very little. Currently, the widely used method to improve the reconstructed image quality is the quadtree-based adaptive partitioned scheme [17]. The key idea of the quadtree FIC scheme is still to reduce the size of range blocks. Firstly, the original image is partitioned into some range blocks with larger size. If one range block can be well approximated by the iterated function systems (IFS), the encoding for the range block is implemented with the current size. If one range block cannot be well approximated, the range block will be partitioned into four smaller blocks, and the encoding phase goes on for these small blocks. An error-threshold needs to be set to judge whether a range block is well approximated.

The quadtree-based FIC scheme, though, can partly improve the reconstructed image quality, it has some fatal shortcomings. Firstly, the original image needs to be partitioned into some range blocks with different sizes in the quadtree scheme, which means that we also need to construct several domain pools with different sizes. Therefore, the quadtree scheme further increase the encoding time of FIC. Secondly, if the number of layers in quadtree scheme is *k*, the side length of the range blocks in the first layer must be divided exactly by 2^*k*−1^. Hence, the adaptability of partition in quadtree scheme is weak. Thirdly, because it is the key idea of quadtree scheme to reduce the block size, the convergence problem is still existed in the quadtree-based FIC scheme.

In this paper, we apply the thinking of sparse coding [21, 22] into FIC technology to propose a sparse fractal image compression (SFIC) scheme, in which the matching process between the range blocks and the domain blocks is performed by the idea of sparse coding instead of the idea of vector quantization. The proposed SFIC scheme can greatly improve the reconstructed image quality of FIC, and it also can effectively overcome the three main shortcomings of the quadtree scheme. Furthermore, because the domain blocks in SFIC are all kept the same size, the proposed SFIC scheme can be perfectly combined with the APCC-based FIC accelerating scheme. The combined scheme is an efficient and effective FIC scheme, and we call it the fast sparse fractal image compression (FSFIC).

The rest of the paper is structured as following. In Section II, we briefly introduce sparse coding and the APCC-based FIC scheme. We analyze the main cause of the low reconstructed image quality in FIC and present the sparse fractal image compression scheme in Section III. The fast sparse fractal image compression scheme is proposed in Section IV. The experiment results and experiment analysis are given in Section V. Finally, we drawn the conclusions in Section VI.

## Sparse coding and APCC-basd FIC

In this paper, we will apply the thinking of sparse coding into FIC, then combine the sparse fractal image compression scheme and the APCC-based FIC scheme to improve both the efficiency and the reconstructed image quality of FIC.

### Sparse coding

Sparse coding (SC) is an important tool in various fields such as pattern recognition [23, 24] and classification [25–29]. It is a bionic image compression method proposed by Olshausen and Field, which mainly simulates the visual processing of the simple cells in mammalian visual cortex. In SC, the image blocks which are used to encode other blocks are called the “basis”. Many dictionary learning technologies had been proposed to train the basis set for different questions [27–30]. Suppose we have an overcomplete basis set **V** = {**v**_1_, **v**_2_, ⋯, **v**_*n*_} in which each **v**_*i*_ is an image block with size *B* × *B*, *i* = 1, 2, ⋯, *n*. The word “overcomplete” means that the number of bases in the basis set is much larger than *m* = *B* × *B*. If all the bases in the overcomplete basis set are zero-meaned, then for an image block **R** with the same size, the sparse coding approximates **R** by a linear combination of the bases as following:

$$\begin{array}{c} R\approx \lambda_{1}v_{1}+\lambda_{2}v_{2}+\cdots +\lambda_{n}v_{n}+\mu_{R}1 \end{array}$$ (1)

where *μ*_**R**_ is the mean of **R**, *λ*_*i*_ is the affine parameter of **v**_*i*_ for **R**, *i* = 1, 2, ⋯, *n*, and **1** is the block with all ones. In SC, only a few of the affine parameters *λ*_*i*_ are non-zero.

When the basis set is given, it is the most important to find these bases with non-zero affine parameters for a block **R** to be encoded. Matching pursuit (MP) [31] and orthogonal matching pursuit (OMP) [32] are two commonly used solutions.

Generally, an error threshold is set in MP. For a zero-meaned block **R**, MP firstly searches a basis **v**_*i*_ from the basis set to minimize the mean square error (MSE) between the target block **R** and *λ*_*i*_ **v**_*i*_, in which *λ*_*i*_ is the affine parameter computed by the least square method. If the value of MSE(**R** − *λ*_*i*_ **v**_*i*_) is smaller than the error threshold, **R** ≈ *λ*_*i*_ **v**_*i*_. If not, MP takes the residual **R** − *λ*_*i*_ **v**_*i*_ as the new target block and go on. Finally, when the *l*-2 norm of the residual is smaller than the given threshold, MP stops and the block **R** is approximated by the addition of all the linear parts. If block **R** is not zero-meaned, it needs only add *μ*_**R**_ **1** in the linear parts.

OMP is a modified MP algorithm. In each step of OMP, the residual is maintained full orthogonality with the former linear parts. Therefore, OMP has better convergence than MP.

### APCC-based FIC scheme

According to the fact that the self-similarity in fractal image compression is Pearson’s absolute correlation, we proposed an APCC-based FIC scheme in 2013 [16].

The first step of the APCC-based scheme is the 3-class method proposed by Fisher [33], which we have proved to be an APCC-based classification method.

In Fisher’s 3-class method, a square block **D** is partitioned into four square sub-blocks, the upper-left square block, the upper-right square block, the lower-left square block, and the lower-right square block. For eight transformations [34]

  *T*_0_: Identity

  *T*_1_: Orthogonal reflection about mid-vertical axis

  *T*_2_: Orthogonal reflection about mid-horizontal axis

  *T*_3_: Orthogonal reflection about first diagonal

  *T*_4_: Orthogonal reflection about second diagonal

  *T*_5_: Rotation around center of block, through +90°

  *T*_6_: Rotation around center of block, through +180°

  *T*_7_: Rotation around center of block, through +270°,

if the luminance sums of the four square sub-blocks vary each other, then there is only one block in *T*_0_(**D**), *T*_1_(**D**), ⋯, *T*_7_(**D**) belonging to one of the following three classes:

$$\begin{array}{c} \begin{array}{c} \text{Class1:}\;a_{1}\geq a_{2}\geq a_{3}\geq a_{4}\\ \text{Class2:}\;a_{1}\geq a_{2}\geq a_{4}\geq a_{3}\\ \text{Class3:}\;a_{1}\geq a_{4}\geq a_{2}\geq a_{3} \end{array}. \end{array}$$ (2)

where *a*_1_, *a*_2_, *a*_3_, and *a*_4_ are the luminance sums of the four square sub-blocks in this transformed block, respectively.

According to Fisher’s 3-class method, the range blocks and the domain blocks are classified into 3 classes, then the matching blocks can be searched in the same class.

In the second step of APCC-based FIC scheme, the domain blocks in each class are sorted by the absolute values of Pearson’s correlation coefficients between these domain blocks and an offline-trained block **B**. For a range block **R**, if the absolute value of Pearson’s correlation coefficient between **R** and **B** is |*r*_**RB**_|, we can then search the matching domain block for **R** in a small domain block set in which the absolute values of Pearson’s correlation coefficients between these domain blocks and **B** is close to |*r*_**RB**_|.

In BFIC, the eight transformed blocks from one partitioned domain block by the eight transformations *T*_0_, *T*_1_, ⋯, *T*_7_ are all included in the domain pool. However, in the APCC-based scheme, only one transformed block is included. Therefore, the number of domain blocks in the APCC-based scheme is only one eighth of that in BFIC. Block classification and block sorting also greatly reduces the encoding time. As a whole, the APCC-based FIC scheme significantly speeds up encoding in FIC.

## Sparse fractal image compression

Low quality of the reconstructed images is one of the main bottlenecks in FIC. In this section, we try to find a solution for this question.

### Problem analysis

In FIC, the range blocks are taken as the blocks to be encoded, and the domain blocks are taken as the codewords. The matching process between the range blocks and domain blocks is, in fact, the searching strategy in vector quantization. For each range block, the matching domain block is searched in all the domain blocks. Then a question appears that if the domain blocks of an image cannot approximate the range blocks well, the quality of the reconstructed image is hard to meet the requirements.

The methods, such as enlarging the domain pooling by decreasing the step-length between two adjoining domain blocks, only improve little of the reconstructed image quality. They are not the essential solution of this question.

By further analysis of the FIC technology, we find that the key point of the image quality problem in FIC is the “dimension”. Suppose the size of the range block is *B* × *B*, then the dimension of the range block is *m* = *B* × *B*. In FIC, if the matching domain block for the range block **R** is **D**, **R** is then approximated by *s***D** + **o1**, where *s* and *o* are the affine scalar parameters computed by the least square method, and **1** is the block with all ones.

Clearly, each *m*-dimensional range block is only expressed by the two vectors **D** and **1** in FIC. It is known that *m* linearly independent vectors can losslessly express one *m*-dimensional target vector. Hence, the dimension is the key factor to improve the reconstructed image quality. From the view of dimension, there are two main methods. It is the first method to reduce the dimension of range block because a vector with lower dimension can be expressed better by two vectors. This method can be accomplished by reducing the partitioned size of range blocks. Actually, the quadtree scheme is one of its concrete models. It is the second method to approximate a range blocks with more domain blocks. Here, we will take the second method into consideration.

### The method

In the baseline fractal image compression, the searching strategy takes use of the idea of vector quantization. Vector quantization approximates a vector by only one codeword and a luminance offset. To approximate a vector with more than one codeword, we need use the searching strategy of sparse coding instead of vector quantization.

As a virtual codebook, the domain pool of FIC usually includes a large number of domain blocks, which can be taken as an overcomplete basis set in sparse coding. Then the matching process between the range blocks and the domain blocks is to search the corresponding basis or bases in domain pool for each range block. Hence, the searching strategy of sparse coding can be directly applied in FIC. We call the novel fractal image compression scheme with the searching strategy of sparse coding the sparse fractal image compression (SFIC). Compared with BFIC, SFIC can code a range block with more than one domain block.

In SFIC, the partition of the original image is kept the same as BFIC. Suppose the size of the range blocks is *B* × *B*, the size of the original domain blocks is 2*B* × 2*B*. Then all the range blocks are constructed the range pool. Subsequently, all the original domain blocks are contracted into size *B* × *B* to keep the same size as the range blocks, and the eight transformations *T*_0_, *T*_1_, ⋯, *T*_7_ are applied to the domain blocks with size *B* × *B* to construct the domain pool. In SFIC, we take the domain pool as the overcomplete basis set in sparse coding, which is only used during encoding but not during the decoding process.

After the range pool and the domain pool are constructed, we find some blocks **D**_*i*_ in the domain pool by the means of MP or OMP for each range block to approximate the range block. Suppose *k* blocks **D**_*i*_ in the domain pool are chosen for each range block, *i* = 1, 2, ⋯, *k*, and the linear coefficients for each domain block are *s*_1_, *s*_2_, ⋯, *s*_*k*_, respectively. Because the range block **R** and the domain blocks **D**_*i*_ are not zero-meaned, we need use the block **1** to eliminate these means. Then we have

$$\begin{array}{ccc} R & \approx & s_{1}D_{1}+s_{2}D_{2}+\cdots +s_{k}D_{k}+o1\\ o & = & \mu_{R}-\left(s_{1}\mu_{D_{1}}+s_{2}\mu_{D_{2}}+\cdots +s_{k}\mu_{D_{k}}\right), \end{array}$$ (3)

where *μ*_**R**_ and *μ*_**D**_*i*__ are the means of **R** and **D**_*i*_, respectively, *i* = 1, 2, ⋯, *k*.

**Fig 1:**
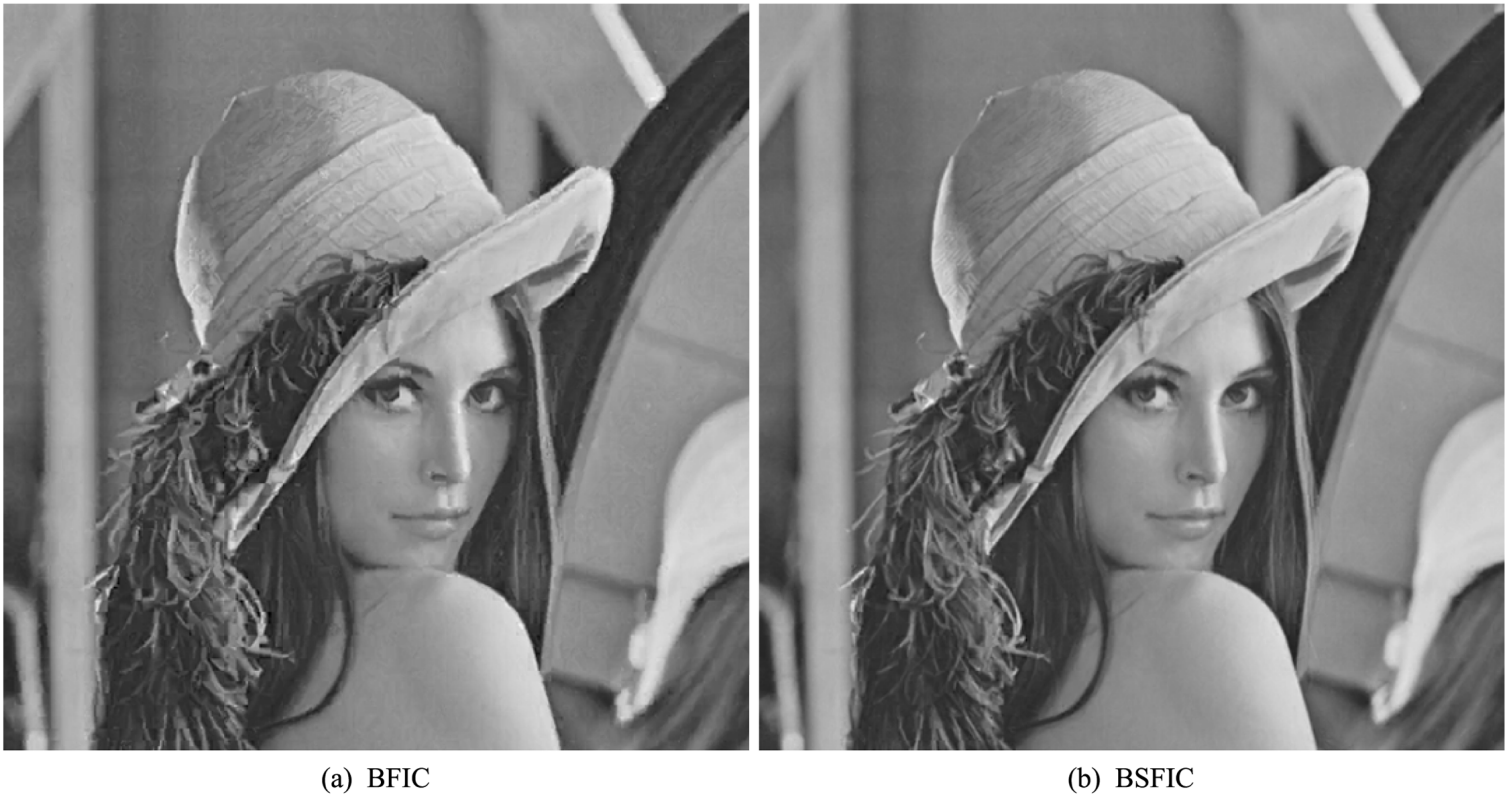
The reconstructed images of 512 × 512 Lena with range size 8 × 8 for BFIC scheme and BSFIC scheme, respectively.

Bilinear sparse fractal image compression (BSFIC) with *k* = 2 in Eq (3) is one of the simplest forms of SFIC. In BSFIC, each range block is approximated by two domain blocks, and the coefficients of the domain blocks can be computed by the bilinear vector quantization [35]. The reconstructed images of 512 × 512 Lena with range size 8 × 8 are shown in Fig 1 for BFIC scheme and BSFIC scheme, respectively. More comparisons of the reconstructed image quality between BFIC scheme and BSFIC scheme are given in Table 1, from which we can see the BSFIC scheme improves the quality of the reconstructed images with 2.38 dB in average. The 13 test images with size 512 × 512 are aerial, baboon, boat, bridge, couple, F16, goldhill, house, Lena, man, milkdrop, pepper, and sailboat, respectively.

**Table 1:** PSNR/dB of the reconstructed images in BFIC scheme and BSFIC scheme.

Images	BFIC	BSFIC	Images	BFIC	BSFIC
aerial	24.21	27.05	house	27.53	30.27
baboon	22.39	24.07	Lena	32.20	35.43
boat	27.85	30.52	man	28.95	31.60
bridge	24.60	26.68	milkdrop	36.79	40.28
couple	27.92	29.39	pepper	31.78	33.83
F16	28.04	29.22	sailboat	27.14	29.33
goldhill	30.06	32.73	**Average**	**28.42**	**30.80**

The storage of SFIC is similar as the storage of BFIC. For the block **R**, the storage of the structure expressed in Eq (3) should include the indexes of the blocks **D**_*i*_ in the domain pool and their linear coefficients. For example,

$$\begin{array}{c} (s_{1},\text{index}\left(D_{1}\right),s_{2},\text{index}\left(D_{2}\right)\cdots ,s_{k},\text{index}\left(D_{k}\right),o), \end{array}$$

where index(**D**_*i*_) is the index of **D**_*i*_ in the domain pool.

In SFIC, the storage of each range block **R** includes *k* affine coefficients *s*_1_, *s*_2_, ⋯, *s*_*k*_, *k* indexes and an offset *o*. If the space occupied by each affine coefficient is *p*, the space occupied by each index is *q*, the space occupied by the offset is *t*, and the space occupied by each pixel in the original image is *l*, then the compression ratio of SFIC is

$$\begin{array}{c} r=\frac{B^{2}l}{k(p+q)+t}. \end{array}$$ (4)

The compression ratio of BFIC is the case of *k* = 1 in Eq (4).

### Adaptive sparse fractal image compression

In quadtree FIC scheme, it can be adaptively decided whether a range block needs to be further partitioned by the error threshold. If a range block has been approximate well with the current size, it does not need the further partition. If the error is still larger than the error threshold, the range block needs to be partitioned into the smaller size.

In SFIC, we can also set a threshold *e* to determine how many domain blocks are needed to approximate a range block. For the range block **R**, we firstly find the matching domain block **D**_1_ and the corresponding linear transformation *s*_1_**D**_1_ + *o*_1_**1**. Let **E**_1_ = **R** − (*s*_1_**D**_1_ + *o*_1_**1**), if ‖**E**_1_‖_2_ ≤ *e* where ‖⋅‖_2_ is the two-norm, we approximate **R** by only one domain block. If ‖**E**_1_‖_2_ > *e*, we encode **R** by more domain blocks until there is one **E**_*k*_ = **R** − (*s*_1_ **D**_1_ + *s*_2_ **D**_2_ + ⋯ + *s*_*k*_ **D**_*k*_ + *o*_*k*_**1**) so that ‖**E**_*k*_‖_2_ ≤ *e*. In practice, we also set a maximum value *k*_1_ for *k*. When *k* > *k*_1_, the encoding process stops.

There is a little difference of the storage between the adaptive sparse fractal image compression (ASFIC) and SFIC. In ASFIC, we need to store the number of domain blocks for each range block as following:

$$\begin{array}{c} \left(k,s_{1},\text{index}\left(D_{1}\right),s_{2},\text{index}\left(D_{2}\right)\cdots ,s_{k},\text{index}\left(D_{k}\right),o\right) \end{array}$$

Here *k* ≤ *k*_1_. The value of *k* may be different for different range blocks in ASFIC.

If the space occupied by storing the number of domain blocks is *a*, and the others are kept the same as that in SFIC, then the compression rate for the range block **R** in ASFIC is

$$\begin{array}{c} r=\frac{B^{2}l}{k(p+q)+t+a}. \end{array}$$ (5)

The compression ratio of a range block in SFIC is a little higher than ASFIC when the value of *k* is kept the same according to Eqs (4) and (5). However, the average number of domain blocks to approximate each range blocks in ASFIC is usually much smaller than the fixed value of *k* in SFIC. Hence, the compression rate of ASFIC for an image is usually much higher than that of SFIC. Generally, we choose ASFIC to encode an image rather than SFIC. Therefore, we still call the adaptive sparse fractal image compression (ASFIC) as the sparse fractal image compression (SFIC) for short.

## Fast sparse fractal image compression

**Fig 2:**
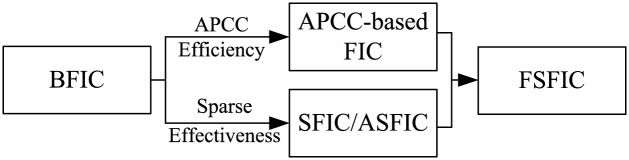
The generating process of the fast sparse fractal image compression (FSFIC) scheme.

Another important problem in fractal image compression is the over-long encoding time. In SFIC, we use the idea of sparse coding instead of the idea of vector quantization to approximate the range blocks, which further increases the encoding time of FIC. In this section, we will propose a fast sparse fractal image compression (FSFIC) scheme by combining the APCC-based FIC scheme and the SFIC scheme. The relation between BFIC, APCC-based FIC, SFIC/ASFIC, and FSFIC is shown in Fig 2.

### The method

Even if one range block is approximated by only one domain block in FIC, the encoding process still suffers from high time complexity. In SFIC, one range block is approximated by more domain blocks, which further decreases the encoding efficiency. Hence, if we have no an effective accelerating method, SFIC will be meaningless.

By analyzing the APCC-based FIC scheme and the sparse fractal image compression scheme, we find that the APCC-based FIC scheme is really a customized method for SFIC. The key steps in the APCC-based FIC scheme is to classify the domain pool according to Fisher’s 3-class method and sort the domain blocks according to the absolute value of Pearson’s correlation coefficients. In SFIC, once the domain pool is constructed, the domain blocks in the domain pool will be totally determined, and the domain blocks are all kept the same size. Hence, the domain pool in SFIC needs to be classified and sorted only once. Then the classified and sorted domain blocks can be directly used to speed up encoding. Hence, we have the steps of the fast sparse fractal image compression (FSFIC) as follows:

#### *a*. Partition

The original image is partitioned with size *B* × *B* to generate the range blocks, and is partitioned with size 2*B* × 2*B* to generate the original domain blocks. Then the original domain blocks are shrunk to kept the same size as the range blocks by averaging four pixels to one pixel.

#### *b*. Classification

By Fisher’s 3-class method, all the shrunk domain blocks are classified into 3 domain classes. For each shrunk domain block **D**, the transformation *T*_**D**_ which transforms **D** into the current class is also saved.

#### *c*. Sorting

For each domain class, the domain blocks are sorted by the absolute value of correlation coefficients between these domain blocks and an offline-trained preset block **B** for this class. Then we obtain three sorted domain classes.

#### *d*. Searching

For each range block **R**, we find the class of **R** and −**R**, and find the transformations *T*_**R**_ and *T*_−**R**_ which transform **R** and −**R** into the same class, respectively. Then two absolute value of Pearson’s correlation coefficients |*r*_*T*_**R**_(**R**)**B**_| and |*r*_*T*_−**R**_(−**R**)**B**_| are computed, which are the APCC between *T*_**R**_(**R**) and **B**, and the APCC between *T*_−**R**_(−**R**) and **B**, respectively. Subsequently, a small domain set in the domain class is chosen with *k*_2_ domain blocks in which the APCCs between the domain blocks and the preset block **B** are close to |*r*_*T*_**R**_(**R**)**B**_|. Then the matching block **D**_**R**_ for *T*_**R**_(**R**) is searched from this small domain set. By the same method, the matching domain block **D**_−**R**_ for *T*_−**R**_(−**R**) is also obtained. Then |*r*_*T*_**R**_(**R**)**D**_**R**__| and |*r*_*T*_−**R**_(−**R**)**D**_−**R**__| are computed. The larger of them is corresponding to the matching domain block for the range block **R** in this class. Without loss of generality, we suppose that *T*_**R**_(**R**) and **D**_**R**_ are the range block and the domain block corresponding to the best matching pair in this class, and **D** is the untransformed shrunk domain block of **D**_**R**_. Then the final matching domain block for the range block **R** is $T_{R}^{-1}T_{D}\left(D\right)$. By the least square method two parameters *s* and *o* can be computed so that the 2-norm of the residual **E** = $R-(sT_{R}^{-1}T_{D}\left(D\right)+o1)$ gets the minimum value.

#### *e*. MP or OMP process

If ‖**E**‖_2_ ≤ *e* or the number of the domain blocks reaches the maximum value *k*_1_, the encoding of **R** is completed. If not, we take **E** as **R** and repeat the step *d*. In OMP, the residual needs to be maintained full orthogonality with the former linear parts. Hence, the linear coefficients of the former linear parts needs to be updated in OMP.

#### *f*. Representation

The linear combination of all the linear parts are used to approximate the block **R**, in which the affine coefficients for each domain block is the coefficient *s* computed in step *d* for MP method or updated in step *e* for OMP method, and the offset *o* can be computed according to Eq (3).

### The storage

The storage of SFIC includes the linear coefficients and the indexes of the domain blocks. According to the steps *d*–*f* of the FSFIC scheme, a range block **R** can be finally approximated as following:

$$\begin{array}{ccc} R & \approx & s_{1}T_{R_{1}}^{-1}T_{D_{1}}\left(D_{1}\right)+s_{2}T_{R_{2}}^{-1}T_{D_{2}}\left(D_{2}\right)+\\ & & \cdots +s_{k}T_{R_{k}}^{-1}T_{D_{k}}\left(D_{k}\right)+o1 \end{array}$$ (6)

where **R**_*i*_ is the residual of the *i*th iteration in the MP or OMP process, **R**_1_ = **R**, and *T*_**R**_*i*__ and *T*_**D**_*i*__ are the transformations which transform **R**_*i*_ and **D**_*i*_ into their classes, respectively, *i* = 1, 2, ⋯, *k*.

According to Eq (6), the storage of FSFIC includes not only the linear coefficients and the indexes of the domain blocks, but also these transformations. We have known that *T*_**R**_*i*__, *T*_**D**_*i*__ ∈ {*T*_0_, *T*_1_, *T*_2_, *T*_3_, *T*_4_, *T*_5_, *T*_6_, *T*_7_}. According to APCC-based FIC scheme, $T_{j}^{-1}T_{k}\in \{T_{0},T_{1},T_{2},T_{3}$, *T*_4_, *T*_5_, *T*_6_, *T*_7_}, *j* = 0, 1, ⋯, 7, *k* = 0, 1, ⋯, 7. Hence, all the 64 cases of $T_{j}^{-1}T_{k}$ can be offline computed. When we store $T_{R_{i}}^{-1}T_{D_{i}}$, we only need to store the corresponding transformation in {*T*_0_, *T*_1_, *T*_2_, *T*_3_, *T*_4_, *T*_5_, *T*_6_, *T*_7_}.

In practise, if the domain pool in decoding only includes these untransformed shrunk domain blocks, we need to store both the transformation and index for each domain block. If the domain pool includes all the domain blocks transformed by the eight transformations as that in BFIC, we need only store the corresponding index in the domain pool for each domain block, which can be derived from the transformation and the index of the untransformed shrunk domain block.

Actually, for the above two different domain pool, although their methods of storage are different, the space occupied by storing the information for each domain block is kept the same. The space occupied by storing a transformation is only 3 bits because there are eight transformations. Suppose the number of the domain blocks in the untransformed domain pool is *m*, then the number of the domain blocks in the transformed domain pool is 8*m*. Obviously, the space to store the transformation and the index of the untransformed domain block is the same as the space to store the index of the transformed domain block.

## Experiment results and analysis

Some experiments are performed in this section to show the performance of the proposed FSFIC scheme. The demos are all implemented in the VC++6.0 environment, and the experimental data are collected from the computer with CPU i3-2100 and windows XP operating system. OMP method is used for the searching of FSFIC.

We use 13 bins to store each affine parameter *s*, and 15 bins to store each offset *o* in Eq (6). If 2^q^ is not less than the number of domain blocks and 2^*q*−1^ is less than the number of domain blocks, *q* bins is then used to store each index of domain blocks.

### Parameter analysis in FSFIC

In FSFIC, the main parameters include the maximum number *k*_1_ of the domain blocks to encode a range block, the number *k*_2_ of the domain blocks in the selected small domain set in the searching step, and the error threshold *e* to determine how many domain blocks are needed for a range block.

Firstly, we discuss the impact of *k*_1_, *k*_2_, and *e* on the reconstructed image quality, the compression ratio, and the encoding time, respectively.

#### *a*. Parameter *k*_1_

In SFIC, *k*_1_ is the key parameter to show the thinking of sparse coding. SFIC with *k*_1_ = 1 is just the baseline FIC.

The actual number of domain blocks to encode a range block is decided not only by the maximum domain block number *k*_1_, but also by the error threshold *e*. There are two conditions to stop the encoding process. One is that the *l*-2 norm of the residual is less than *e*, and the other is that the number of domain blocks in the linear combination to approximate the range block reaches *k*_1_.

**Fig 3:**
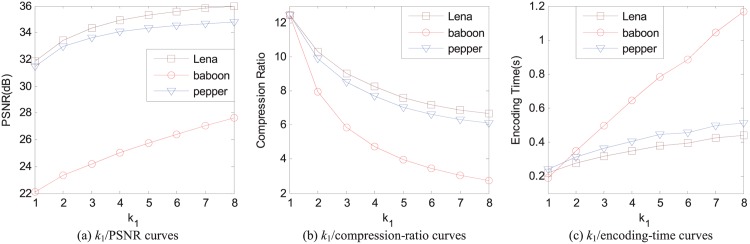
The relations of different values of *k*_1_ with the reconstructed image quality, the compression ratio, and the encoding time for 512 × 512 images Lena, baboon, and pepper, respectively. The range size is 8 × 8, *k*_2_ is the number of all the domain blocks in the domain pool, and the average pixel-based square error *e* = 25.

The impact of *k*_1_ on the reconstructed image quality, the compression ratio, and the encoding time is shown in Fig 3 for three 512 × 512 images Lena, baboon, and pepper, respectively. The range size is 8 × 8, the parameter *k*_2_ is set as the number of domain blocks in the domain pool, and the error threshold *e* = 25 so that the average pixel-based square error is less than 25.

We can see from Fig 3a that the values of PSNR of the reconstructed images for each image increase when the value of *k*_1_ increases, which is one of the main reasons why we apply the thinking of sparse coding into the baseline FIC. It is also can be seen from Fig 3a that the incremental of PSNR value is gradually decreased when the value of *k*_1_ increases. In practice, if the value of *k*_1_ is too small, the image quality may not meet the requirements. However, too large value is also a bad choice because the marginal effect of the maximum value *k*_1_ is gradually decreased. Hence, the value of *k*_1_ should be properly set according to the actual demands.

The curves of the compression ratio of images against *k*_1_ are shown in Fig 3b for images Lena, baboon, and pepper, respectively. The compression ratio is also decreased when *k*_1_ increases. Hence, when we encode an image in SFIC, we should find a balance in the reconstructed image quality and the compression rate.

In Fig 3c, the encoding time curves are given for three images Lena, baboon, and pepper with different values of *k*_1_. Although here we set *k*_2_ the number of domain blocks in the domain pool, the encoding speed is still much faster than the baseline FIC. We can also see that there is an approximate linear relation between the encoding time and *k*_1_.

From Fig 3a–3c, *k*_1_ has a big impact on the reconstructed image quality, the compression ratio, and the encoding time. Thus, *k*_1_ is a key parameter in SFIC. We should pay more attention to carefully choose the value of *k*_1_ to meet different requirements.

#### *b*. Parameter *k*_2_

**Fig 4:**
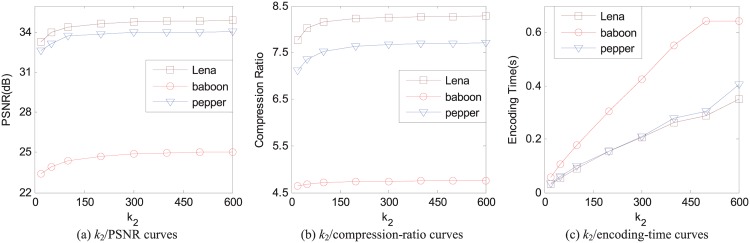
The relations of different values of *k*_2_ with the reconstructed image quality, the compression ratio, and the encoding time for 512 × 512 images Lena, baboon, and pepper, respectively. The range size is 8 × 8, *k*_1_ = 4, and the average pixel-based square error *e* = 25.

In the APCC-based FIC scheme, *k*_2_ is the number of domain blocks in the selected domain set to encoding the corresponding range block. The main function to select the domain set is to speed up the encoding process. A small *k*_2_ can significantly accelerate the encoding of FIC while preserving the reconstructed image quality well. In SFIC, *k*_2_ also has its impact on the reconstructed image quality, the compression ratio, and the encoding time, as shown as in Fig 4, in which *k*_1_ = 4 and the error threshold *e* = 25 so that the average pixel-based square error is less than 25.

The relations between *k*_2_ and the reconstructed image quality for images Lena, baboon, and pepper are shown in Fig 4a, respectively. When the value of *k*_2_ is selected below 100, the PSNR values of the reconstructed images get a big change. When the value of *k*_2_ is over 300, the PSNR values of the reconstructed images almost keep invariable. Therefore, we can choose *k*_2_ in the interval [100, 300] to speed up the encoding while preserving the reconstructed image quality well.

The shape of the curves of the compression ratio against *k*_2_ given in Fig 4b is similar as the curves of the reconstructed image quality against *k*_2_. In the interval [0, 100] of the value of *k*_2_, the compression ratio changes largely. In the interval [300, +∞] of *k*_2_, the compression ration keep almost invariable. As we can see from Fig 4a and 4b, both the compression ratio and the reconstructed image quality increase when the value of *k*_2_ increases.

Like the relation between the encoding time and *k*_1_, the encoding time and *k*_2_ also keep an approximate linear relations, as shown in Fig 4c.

According to these discussions about *k*_2_, we should choose *k*_2_ properly to reach an optimum result. For example, the interval [100, 300] is a good choice for *k*_2_ to encode a 512 × 512 image. The encoding time in Fig 4c is less than 0.2 seconds with *k*_1_ = 4 and *k*_2_ = 100, and is about 0.2 to 0.4 seconds with *k*_1_ = 4 and *k*_2_ = 300.

#### *c*. Parameter *e*

The error threshold *e* is used to decide the actual number of domain blocks to approximate one range block. If the *l*-2 norm of the residual is less than *e* for one range block, the encoding stops.

**Fig 5:**
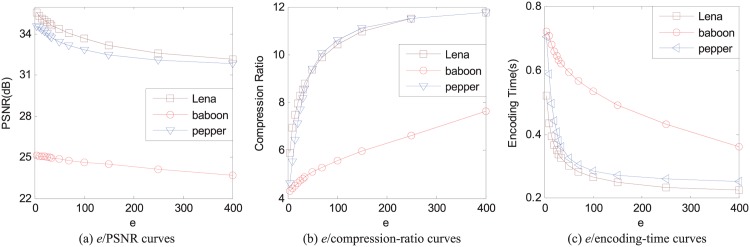
The relations of different values of the average pixel-based square error *e* with the reconstructed image quality, the compression ratio, and the encoding time for 512 × 512 images Lena, baboon, and pepper, respectively. The range size is 8 × 8, *k*_1_ = 4, and *k*_2_ is the number of all the domain blocks in the domain pool.

The impact of *e* on the reconstructed image quality, the compression ratio, and the encoding time is shown in Fig 5, respectively, with *k*_1_ = 4 and *k*_2_ the number of domain blocks in the domain pool.

A small *e* can improve the reconstructed image quality according to Fig 5a. However, small *e* greatly increases the encoding time and decreases the compression ratio according to Fig 5b and 5c. Hence, we have to choice the value of *e* to make an acceptable compromise in the reconstructed image quality, the compression ratio, and the encoding time. For example, we set the average pixel-based square error as 25 in Figs 3 and 4.

#### *d*. Parameters *k*_1_ and *e*

**Fig 6:**
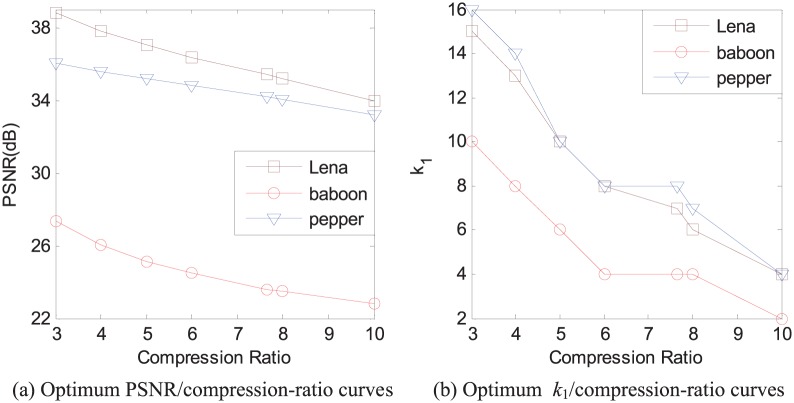
The optimum cases of the reconstructed image quality for different compression ratios with fixing *k*_2_ as the number of all the domain blocks in the domain pool.

Here we fix *k*_2_ as the number of all the domain blocks in the domain pool to construct the optimum combinations of the reconstructed image quality and the compression ratios for 512 × 512 images Lena, baboon, and pepper with range size 8 × 8, as shown in Fig 6.

In Fig 6a, the optimum curves of PSNR against the compression ratio are given for images Lena, baboon, and pepper, respectively. When the compression ratio is 10, the values of PSNR of the reconstructed images Lena, baboon, and pepper are 33.97 dB, 22.83 dB, and 33.20 dB, respectively. When the compression ratio is 5, the values of PSNR of the reconstructed images Lena, baboon, and pepper are 37.03 dB, 25.13 dB, and 35.16 dB, respectively.

Compared with Fig 6a and Table 1, we can see that the bilinear sparse fractal image compression (BSFIC) and FSFIC have their own advantages in the reconstructed image quality. In the BSFIC scheme, the compression ratio is always 7.6418 when these parameters are occupied the same space as we introduced in the front of this section. The values of PSNR in BSFIC are 35.43 dB, 24.07 dB, and 33.83 dB for three 512 × 512 images Lena, baboon, and pepper, respectively. When the compression ratio is also 7.6418 in the proposed FSFIC schemes, the corresponding values of PSNR in the FSFIC scheme are 35.40 dB, 23.59 dB, and 34.18 dB, respectively. However, the encoding time in the BSFIC scheme is far more than the encoding time in the FSFIC scheme because each pair of domain blocks must be considered for each range block. In our experiment environment, the time to encode one 512 × 512 images with range size 8 × 8 in the BSFIC is over 3 minutes.

According to Fig 6b, in the optimum cases of the combinations of the reconstructed image quality and the compression ratio, *k*_1_ gradually decreases when the compression ratio increases, which is well fit with our intuition. For other images, Fig 6b also offers a reference for how to properly choose the corresponding values of *k*_1_ with different compression ratios.

### Efficiency analysis of FSFIC

Efficient encoding method plays an important role in the SFIC scheme. The fast SFIC scheme proposed in this paper takes use of the APCC-based classification and sorting method to accelerate the encoding process of SFIC. We had shown that the APCC-based classification and sorting method can significantly speed up FIC [16]. Here we discuss the performance of the APCC-based classification and sorting method on SFIC.

**Fig 7:**
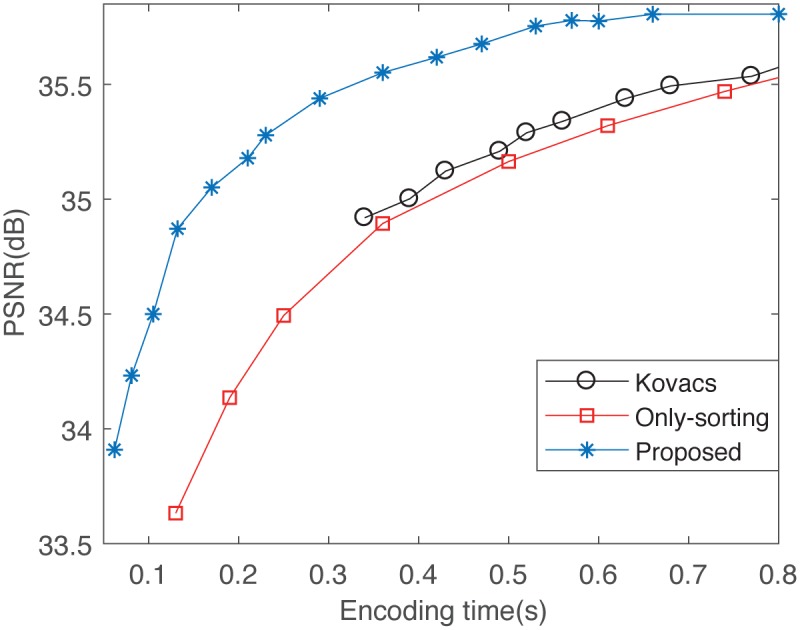
The comparisons among these SFIC schemes with different accelerating technologies on 512 × 512 image Lena with sparse number 4. These accelerating technologies include the APCC-based classification and sorting method (the proposed FSFIC scheme), the APCC-based only sorting method (only-sorting), the polar angle and NRMS-based method (Kovacs), and the hog feature-based method. Because the encoding time of the HOG-based method is always more than 1.5*s* in our experiment, it doesn’t appear in this figure.

We implemented sevveral existing accelerating methods with c++ in the SFIC scheme, including the hog feature-based method [36], the polar angle and the normalized root mean square error (NRMS)-based method [37], and the APCC-based only sorting method [16]. The comparisons among these SFIC schemes with different accelerating technologies are given in Fig 7.

From Fig 7 we can see that when the encoding time is about 0.75*s*, the PSNR values of the decoded images are about 35.5dB in the only-sorting scheme and the Kovacs’s scheme. However, in the proposed FSFIC scheme, it only spend about 0.3*s* to achieve the decoded images whose PSNR value is about 35.5dB. Some other methods, such as the hog feature-based method, even cannot encode the 512 × 512 image Lena with sparse number 4 within 1.5*s*. Therefore, the proposed FSFIC scheme is an efficient SFIC technology.

### Quadtree-based scheme and FSFIC scheme

From the above experiments we can see that the proposed FSFIC scheme can greatly improve the reconstructed image quality compared with the BFIC scheme. Moreover, the proposed scheme is much more efficient than the BFIC scheme. Next we compare the proposed FSFIC scheme with the quadtree-based FIC scheme.

Both the quadtree-based FIC scheme and the proposed FSFIC scheme can improve the reconstructed image quality in FIC. The former improves the reconstructed image quality by decreasing the range size, and the later improves the reconstructed image quality by increasing the number of domain blocks to encode a range block. However, three important limitations exist in the quadtree-based FIC scheme. They are the overlong time in encoding, the limitation of the side length of the range blocks, and the low convergence of decoding. Below we will discussion these shortcomings of the quadtree-based FIC scheme and show the performance of the proposed FSFIC scheme when it faces the same problems.

#### Encoding time

In the quadtree-based FIC scheme, we have to construct several domain pools with different sizes to encode the range blocks with different sizes. Furthermore, in the encoding process of the quadtree-based FIC scheme, if a range block is finally encoded by a domain pool with a small size, the range block has definitely been encoded by all the domain pools with larger sizes, and all the works for this range block performed on these domain pools with larger sizes are discarded. Hence, the quadtree-based FIC scheme usually takes much more time than the baseline FIC to encode an image. In our experimental environment, it spends 18.3 seconds to encode the 512 × 512 image Lena with the largest range size 16 × 16, the smallest range size 4 × 4, and the average pixel-based square error threshold 25.

The proposed FSFIC scheme can encode an image with high efficiency because the APCC-based speedup strategy is used, which can significantly accelerate the encoding process while preserving the reconstructed image quality well. Moreover, the MP or OMP-based method can be perfectly combined with the APCC-based strategy. The actual encoding time has been provided in Figs 3c, 4c and 5c for 512 × 512 images Lena, baboon, and pepper, respectively, with the range size 8 × 8 in the proposed FSFIC scheme, which is much less than that in the quadtree-based FIC scheme.

If the number of layers in the quadtree scheme is *k*, the side length of the range blocks in the first layer must be divided exactly by 2^*k*−1^ and the side length of the domain blocks in the first layer must be divided exactly by 2^k^. However, in the proposed SFIC scheme, the side length of the range blocks can be set as any positive even numbers. The positive odd numbers cannot be used as the range size because each range block needs to be partitioned into four small square blocks in the Fisher’s 3-class method. Hence, from the views of range size, SFIC is more adaptable than the quadtree-based FIC scheme.

#### Convergence

Although we can set the absolute value of the affine coefficients less than 1 to guarantee the convergence of the iterated function systems (IFS), it may seriously degrade the quality of the reconstructed images by intervening the value of the affine coefficients. Hence, the convergence of the reconstructed images without setting the absolute value of each affine coefficient less than 1 is a very important feature in FIC.

In BFIC, the convergence of IFS weakens with decreasing the range size. When the range size is set as 16 × 16, almost all the IFSs for different images are convergent. However, the convergence of IFSs for lots of images becomes weaker when the size of the range blocks is 4 × 4 or 2 × 2. The same problem appears in the quadtree-based FIC scheme because the quadtree FIC is, in fact, an adaptive BFIC method. If the minimum range size is 4 × 4 or 2 × 2 in the quadtree-based FIC scheme, the reconstructed images for lots of images are not well convergent.

**Fig 8:**
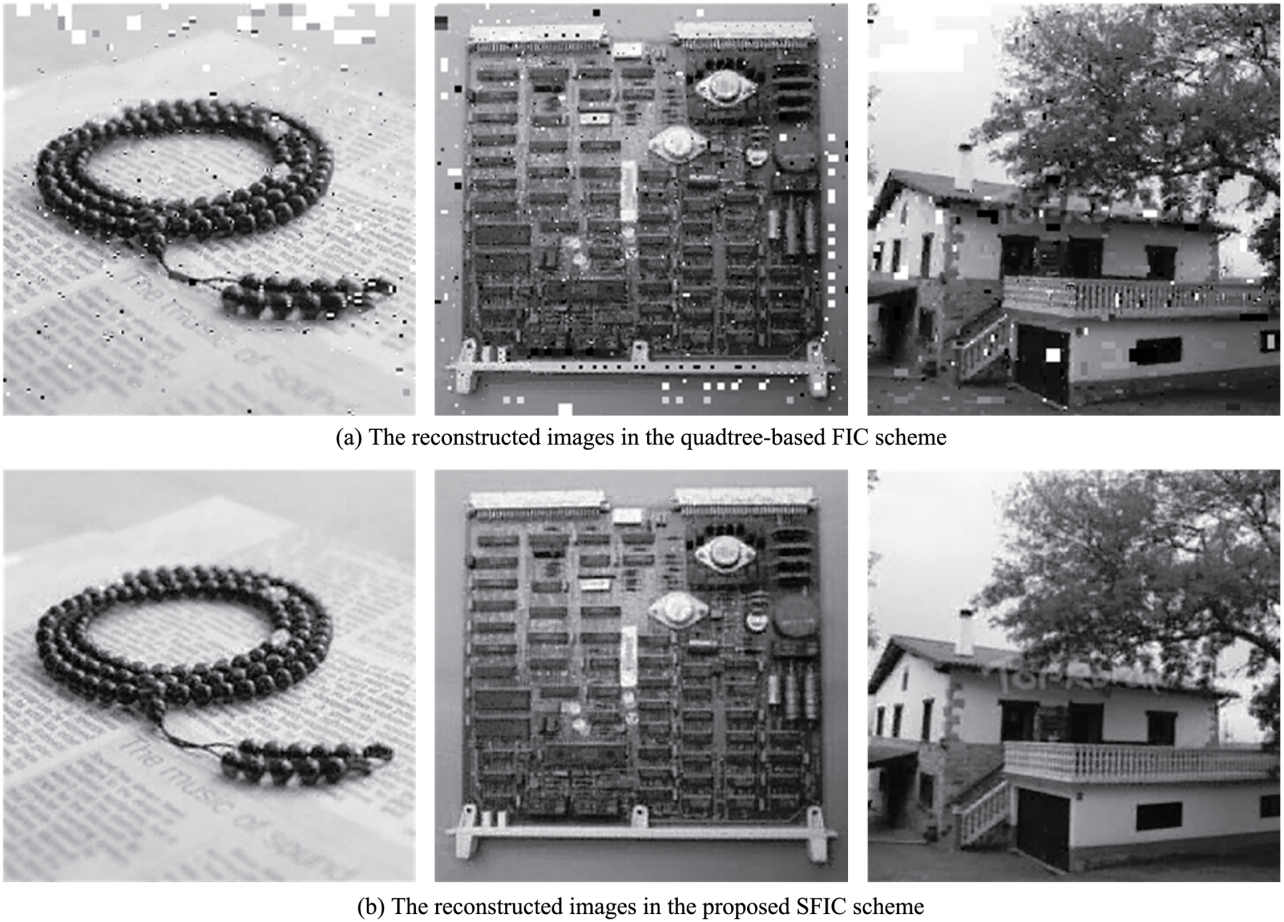
Three examples to show the advantage of convergence of the proposed FSFIC scheme. The images in the first line are the reconstructed images in the quadtree FIC scheme, and the images in the second line are the reconstructed images in the proposed FSFIC scheme.

Several examples are provided in Fig 8 in which the reconstructed images in the quadtree-based FIC scheme have some convergence problems but they are all well convergent in the proposed SFIC scheme. The experiments show that the proposed SFIC scheme can greatly improve the convergence of the reconstructed images. More experiments show that the convergent reconstructed images in the quadtree-based FIC scheme are also convergent in the proposed SFIC scheme.

#### Adaptability of partition

If the number of layers in the quadtree scheme is *k*, the side length of the range blocks in the first layer must be divided exactly by 2^*k*−1^ and the side length of the domain blocks in the first layer must be divided exactly by 2^k^. However, in the proposed SFIC scheme, the side length of the range blocks can be set as any positive even numbers. The positive odd numbers cannot be used as the range size because each range block needs to be partitioned into four small square blocks in the Fisher’s 3-class method. Hence, from the views of block size, SFIC is more adaptable than the quadtree-based FIC scheme.

According to these discussions, the proposed FSFIC scheme can effectively overcome three main disadvantages existed in the quadtree-based scheme.

## Conclusion

The two main problems facing fractal image compression (FIC) technology are the over-long encoding time and the poor reconstructed image quality for some images with low affine similarity. Based on the affine similarity in FIC is equivalent to Pearson’s absolute correlation, we proposed the APCC-based FIC scheme in 2013, which significantly speed up encoding in FIC while preserving the reconstructed image quality of BFIC well. In this paper, we proposed a sparse fractal image compression (SFIC) scheme to approximate each range block by more domain blocks, which can greatly improve the reconstructed images quality in FIC and greatly enhance the convergence of the iterated function system. Moreover, the APCC-based FIC scheme can be perfectly combined into the SFIC scheme. Finally, the fast sparse fractal image compression (FSFIC) scheme was proposed. The experimental results shows that the proposed SFIC scheme can not only significantly speed up encoding, but also greatly improve the reconstructed images quality in FIC.
